# Supplementary material for: Protein domain-based prediction of drug/compound–target interactions and experimental validation on LIM kinases
Source: PLoS Comput Biol. 2021 Nov 29;17(11):e1009171. doi: 10.1371/journal.pcbi.1009171 (PMC8659301; doi:10.1371/journal.pcbi.1009171)
Supplement: S1 Text — (PDF) [file pcbi.1009171.s001.pdf]

S1. Chemical Synthesis of Inhibitor Molecules

S1.1. Synthesis of pyrimidine-based structures **1** and **2** (LIMKi-1 and LIMKi-1a)

Procedure A:

To a solution of 2-chloropyrimidine (10 mmol) and ethyl isonipecotate (10 mmol) in MeCN (5 mL) was added solid potassium carbonate (11 mmol). The resulting reaction mixture was heated at 80 °C for 16 hours. After cooling to ambient temperature and evaporation of acetonitrile the residue was redissolved in ethyl acetate (25 mL) and extracted with water (3 x 10 mL). The organic extract was dried over anhydrous sodium sulfate, filtered and evaporated to dryness to yield the crude ester product as brown liquid (quantitative yield).

The ester intermediate was dissolved in a mixture of water and methanol (50 mL, 1:1 ratio by volume) and treated with solid sodium hydroxide (1.0 g). After heating this mixture at 60 °C for 3 hours, the reaction mixture was allowed to cool to room temperature. The mixture was extracted twice with dichloromethane (2 x 10 mL), the aqueous layer was acidified (1 M HCl) and extracted with dichloromethane (2 x 10 mL). The combined layers of this last extraction were dried over anhydrous sodium sulfate, filtered and evaporated to dryness yielding the corresponding carboxylic acid as colorless oil (92% yield – two steps).

A sample of the carboxylic acid (4 mmol) was dissolved in dry MeCN (1 M solution) and 1,1'-carbonyldiimidazole (5 mmol) was added. After heating for 2 hours at 50 °C the mixture was split into two equal volumes and treated separately with either 3-methylaniline (2.2 mmol) or 3-fluoro-4-methylaniline (2.2 mmol). Each sample was heated at 50 °C for a further 3 hours and the mixtures then allowed to cool to room temperature leading to precipitation of the desired products. Filtration of these solids followed by recrystallization from dichloromethane furnished the desired products (LIMKi-1 and LIMKi-1a) in high yield and purity as white solids.

***N*-(3-Fluoro-4-methylphenyl)-1-(pyrimidin-2-yl)piperidine-4-carboxylate, 1 (LIMKi-1):**

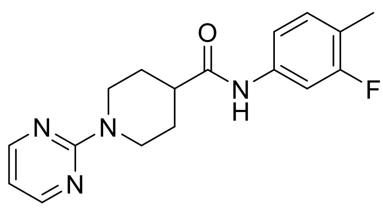

White solid, 83% yield. ^{1}H NMR (400 MHz, Chloroform-*d*) δ 8.28 (d, *J* = 4.7 Hz, 2H), 7.66 (s, 1H), 7.43 – 7.35 (m, 1H), 7.10 – 7.00 (m, 2H), 6.46 (t, *J* = 4.7, 4.7 Hz, 1H), 4.85 – 4.75 (m, 2H), 2.89 (ddd, *J* = 13.4, 12.1, 2.8 Hz, 2H), 2.48 (tt, *J* = 11.6, 3.8 Hz, 1H), 2.19 (d, *J* = 2.0 Hz, 3H), 1.99 – 1.90 (m, 2H), 1.85 – 1.68 (m, 2H). ^{13}C NMR (101 MHz, Chloroform-*d*) δ 173.1 (C), 161.5 (C), 161.0 (CF, d, *J* = 245 Hz), 157.7 (2CH), 136.9 (C, d, *J* = 11 Hz), 131.3 (CH, d, *J* = 6 Hz), 120.6 (C, d, *J* = 18 Hz), 115.1 (CH, d, *J* = 3 Hz), 109.8 (CH), 107.4 (CH, d, *J* = 27 Hz), 44.6 (CH), 43.3 (2 x CH_2),

28.5 (2 x CH_2), 14.1 (CH_3, d, J = 3 Hz). ^{19}F NMR (376 MHz, Chloroform-*d*) δ -115.4. HRMS (TOF ES+) calculated for $C_{17}H_{20}N_4OF$ 315.1621, found 315.1625 (Δ = 1.3 ppm).

1-(Pyrimidin-2-yl)-*N*-(*m*-tolyl)piperidine-4-carboxamide, 2 (LIMKi-1a):

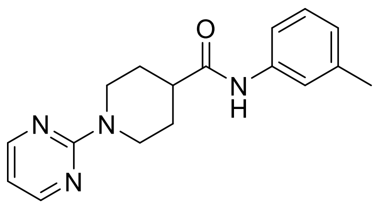

White solid, 79% yield. ^{1}H NMR (400 MHz, Chloroform-*d*) δ 8.28 (d, J = 4.7 Hz, 2H), 7.68 (s, 1H), 7.38 (s, 1H), 7.27 (d, J = 7.8 Hz, 1H), 7.15 (t, J = 7.8 Hz, 1H), 6.94 – 6.84 (m, 1H), 6.45 (t, J = 4.8 Hz, 1H), 4.80 (dt, J = 13.4, 2.7 Hz, 2H), 2.88 (ddd, J = 13.4, 12.1, 2.8 Hz, 2H), 2.48 (tt, J = 11.5, 3.8 Hz, 1H), 2.27 (s, 3H), 1.98 – 1.88 (m, 2H), 1.86 – 1.70 (m, 2H). ^{13}C NMR (101 MHz, Chloroform-*d*) δ 173.1 (C), 161.5 (C), 157.7 (2CH), 138.9 (C), 137.8 (C), 128.8 (CH), 125.2 (CH), 120.7 (CH), 117.1 (CH), 109.8 (CH), 44.6 (CH), 43.3 (2 x CH_2), 28.5 (2 x CH_2), 21.5 (CH_3). HRMS (TOF ES+) calculated for $C_{17}H_{21}N_4O$ 297.1715, found 297.1720 (Δ = 1.7 ppm).

S1.2. Synthesis of thiadiazole-based structures **3** and **4** (LIMKi-2 and LIMKi-3)

Procedure B:

To a suspension of the desired benzamidine hydrochloride hydrate (9 mmol) in dichloromethane (15 mL, 0 °C) was added trichloromethyl sulfenylchloride (10 mmol) and aqueous sodium hydroxide solution (9 mL, 6 N). After stirring this mixture for 1 hour at 0 °C the aqueous layer was separated and piperazine (20 mmol) was added to the organic layer. The resulting mixture was stirred at ambient temperature for 12 hours after which water (20 mL) was added. Extraction of the mixture was performed with dichloromethane (3 x 10 mL) and the combined organic layers were dried over anhydrous sodium sulfate, filtered and evaporated to yield the desired piperazine adduct as an off-white solid (75% yield).

Solutions of the above piperazine adduct were prepared in two separate vials (2 mmol each) in dichloromethane (3 mL each). To each vial was added the corresponding isocyanate (e.g., 3-methylphenylisocyanate or 3-methoxyphenylisocyanate; 2.2 mmol). After stirring this mixture for 5 hours at ambient temperature a white precipitate formed that was isolated by filtration. Recrystallisation from dichloromethane/hexane (1:1) furnished the desired adducts (LIMKi-2 and LIMKi-3) as white solids.

Further members of this small library (e.g. LIMKi-2a-d) were prepared in an analogous fashion and used after appropriate purifications.

4-(3-Phenyl-1,2,4-thiadiazol-5-yl)-*N*-(m-tolyl)piperazine-1-carboxamide, 3 (LIMKi-2):

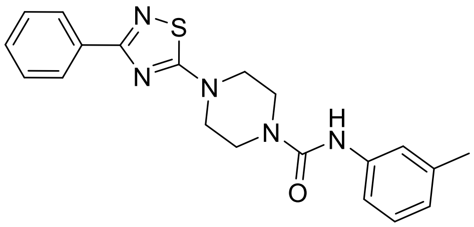

White solid, 60% yield. ^{1}H NMR (700 MHz, DMSO-d_6) δ 8.63 (s, 1H), 8.13 – 8.07 (m, 2H), 7.45 (m, 3H), 7.28 (d, *J* = 2.0 Hz, 1H), 7.25 (dd, *J* = 8.1, 2.2 Hz, 1H), 7.10 (t, *J* = 7.8 Hz, 1H), 6.76 – 6.72 (m, 1H), 3.65 – 3.55 (m, 8H), 2.23 (s, 3H). ^{13}C NMR (176 MHz, DMSO-d_6) δ 185.2 (C), 169.5 (C), 155.3 (C), 140.7 (C), 137.8 (C), 133.3 (C), 130.5 (CH), 129.1 (2 x CH), 128.6 (CH), 128.0 (2 x CH), 123.1 (CH), 120.7 (CH), 117.3 (CH), 48.8 (2 x CH_2), 43.4 (2 x CH_2), 21.6 (CH_3). HRMS (TOF ES+) calculated for $C_{20}H_{22}N_5OS$ 380.1545, found 380.1532 (Δ = 3.4 ppm).

***N*-(3-Methoxyphenyl)-4-(3-phenyl)-1,2,4-thiadiazol-5-yl)piperazine-1-carboxamide, 4 (LIMKi-3):**

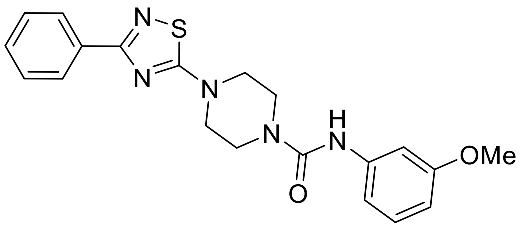

White solid, 66% yield. ^{1}H NMR (700 MHz, Chloroform-*d*) δ 8.20 – 8.15 (m, 2H), 7.46 – 7.38 (m, 3H), 7.18 (t, *J* = 8.1 Hz, 1H), 7.07 (t, *J* = 2.3 Hz, 1H), 6.87 (ddd, *J* = 8.0, 2.1, 0.9 Hz, 1H), 6.78 (d, *J* = 3.5 Hz, 1H), 6.61 (ddd, *J* = 8.3, 2.5, 0.9 Hz, 1H), 3.76 (s, 3H), 3.63 (dd, *J* = 7.2, 3.8 Hz, 4H), 3.62 – 3.58 (m, 4H). ^{13}C NMR (176 MHz, Chloroform-*d*) δ 185.1 (C), 170.4 (C), 160.2 (C), 154.9 (C), 139.9 (C), 133.2 (C), 130.0 (CH), 129.6 (CH), 128.5 (2 x CH), 128.0 (2 x CH), 112.5 (CH), 109.2 (CH), 106.3 (CH), 55.3 (CH_3), 48.3 (2 x CH_2), 43.3 (2 x CH_2). HRMS (TOF ES+) calculated for $C_{20}H_{22}N_5O_2S$ 396.1494, found 396.1490 (Δ = 1.0 ppm).
