# Supplementary material for: Protein domain-based prediction of drug/compound–target interactions and experimental validation on LIM kinases
Source: PLoS Comput Biol. 2021 Nov 29;17(11):e1009171. doi: 10.1371/journal.pcbi.1009171 (PMC8659301; doi:10.1371/journal.pcbi.1009171)
Supplement: S2 Text — (PDF) [file pcbi.1009171.s002.pdf]

S2. Methodological Details of *In vitro* Experimental Assays

All LIMKi (LIM-Kinase Inhibitor) compounds were dissolved in DMSO and stored at -20 ^{0}C as 20 mM stocks.

S2.1. Cell Culture

Human hepatocellular carcinoma cell lines (Huh7, Mahlavu), colon carcinoma cell line (HCT116), breast cancer cell line (MCF-7) were maintained in Dulbecco's Modified Eagle Media (DMEM) (Gibco, Cat:31885-023): together with 10% FBS (Gibco, Cat:10270), 1% Non-essential Amino Acid (MEM-NEAA) (Gibco, Cat:11140-050) and 1% Penicillin-Streptomycin (Gibco, Cat:15140-122); whereas human embryonic kidney cell line (HEK-293) was maintained in same reagents described above together with 100 μg/ml Hygromycin B (Invitrogen, Cat: 10687-010) at 37^{0}C under 5% CO_4. All cells used in this study are STR authenticated and regularly tested for contamination with the mycoplasma test kit (MycoAlert, Lonza, Cat:LT07-118).

S2.2. SRB (Sulforhodamine B) Assay

Cells were collected with trypsinization after being washed with PBS once. Collected cells seeded in 96-well cell culture plate, adjusted with 150 ul/well as followed; Huh-7 (2500 cells/well), Mahlavu (1500 cells/well), HCT-116 (2000 cells/well), MCF-7 (2000 cells/well) and Hek-293 (3000 cells/well). LIMKi compounds were administered in the range of concentration from 40 μM to 2,5 μM, 24 hours later from the initial seeding step. After 72 hours of treatment, cells were fixed with 10% trichloroacetic acid (TCA;Sigma, Cat:27242) and proteins were stained with 0,4% sulforhodamine B sodium salt (SRB; Sigma, Cat: S1402) solution, dissolved in 1% acetic acid (Sigma, Cat: 27225). Plates were read on BMG SpectroStar Nano Spectrophotometer at 515nm. IC_{50} values were calculated based on the normalization according to DMSO-treated (Sigma, Cat: D2650) groups.

S2.3. Western Blotting

500.000 cells of Huh7 and 250.000 cells of Mahlavu were seeded in 150 mm cell culture dishes (Sarstedt, Cat: 83.3903). After 24 hours, the old media was removed and fresh media containing 20 μM of each LIMK inhibitor were added. All treatments were performed as duplicates for 48 hours. At the end of the treatment, cells were scraped and protein extraction was performed. Protein Electrophoresis (Bio-Rad, Mini-PROTEAN Tetra Cell Systems and TGX precast gels) and transfer system (Bio-Rad, Trans-Blot Turbo Transfer System) were used according to the manufacturer's protocol. Proteins were transferred to a PVDF-LF membrane (Bio-Rad, Cat:1620260) Following antibodies were used as described within western blotting protocol. phospho-Cofilin (CST, Cat: 3313) (1:200 v/v), Total Cofilin

(CST, Cat:5175) (1:200 v/v), and IRDye 800CW Goat-anti-Rabbit IgG Secondary Antibody (LI-COR, Cat:926-32211) (1:20000 v/v). For normalization, REVERT 700 Total Protein Stain Kit (LI-COR, Cat:926-11016) was used according to the manufacturer's protocol. Images were taken with LI-COR Odyssey Clx Imaging Device. Signal normalization was performed based on the REVERT Total Protein Stain Normalization protocol by LI-COR Biosciences and imaging analysis was performed by LI-COR, Image Studio Lite software. For efficiency testing for LIMKi compounds with IC_{100} dosages; anti-rabbit IgG (Sigma, Cat: A6154) was used as a secondary antibody (1:5000 v/v), and for imaging; SuperSignal West Femto (Thermo Scientific; Cat: 34095) was used. Imaging was acquired by using LI-COR C-DiGit Blot Scanner. Signal intensity analysis was performed by LI-COR, Image Studio Lite software.

S2.4. Scratch Assay

Huh7 (150.000 cells) and Mahlavu (100.000 cells) cells were seeded to 35 mm cell culture dishes (Corning, Cat:430165) and incubated for at least 24h until cells attached and became confluent. The wound was created in confluent (nearly 100%) monolayer cells by using p30 pipet tip followed by washing with PBS (Gibco, Cat: 14190-169) three times before adding the serum-free medium (1% FBS) that includes LIMK inhibitors or vehicle DMSO. The migration rate of LIMK inhibitor-treated cells was analyzed by comparing samples with the migration of control cells treated with DMSO controls. Gap closure was analyzed by capturing images with time-lapse Nikon ECLIPSE Ti-S inverted microscopy for 10 min intervals for 10 hours (high-quality images of the treated cells are given in the data repository of the study). Upon 10 hours the distance of the same reference point measured at the first and last frame were compared by using NIS-Elements software.

S2.5. Real-Time Cell invasion Analysis

Cells were seeded on CIM-Plate 16 (ACEA, Cat: 05 665 817 001), (20.000 cells/well for Mahlavu and 50.000 cells/well for Huh7 as triplicates) and monitored their invasion capacity on xCELLigence DP RTCA System, in the presence of 20 μM LIMKi compounds. The lower chamber of CIM-Plate was filled with 160 μl DMEM containing 10% FBS. Cells were resuspended with LIMKi compounds in serum-free DMEM (1% FBS, 1% NEAA, and 1% Penicillin / Streptomycin) and inoculated into the upper chamber in 150 ul as final volume. After the inoculation, CIM-Plate was incubated at room temperature for 30 min to allow the cells to settle; then the system was initiated to record CI data for 48 hours with 15-minute intervals. CI values were used to represent time-dependent invasion patterns of cells.

S2.6. Statistical Analysis

All SRB and migration data in this study were obtained from three independent experiments with n ≥ 3 biological replicates. Western Blot experiments were performed as duplicates with 3 independent experiments. The statistical analysis for Western Blot was performed using Welch's *t-test* (Prism, GraphPad) and for the migration assay, Two-way ANOVA (Prism, GraphPad) was performed. Standard deviations of IC_{50} results from SRB Assay and from real-time cell proliferation data were calculated on Microsoft Excel. Statistically significant results were represented as follows: *: p-value <0.05; **: p-value <0.01; ***: p-value <0.001; and ****: p-value <0.0001.
