# Supplementary material for: Protein domain-based prediction of drug/compound–target interactions and experimental validation on LIM kinases
Source: PLoS Comput Biol. 2021 Nov 29;17(11):e1009171. doi: 10.1371/journal.pcbi.1009171 (PMC8659301; doi:10.1371/journal.pcbi.1009171)
Supplement: S3 Text — (PDF) [file pcbi.1009171.s003.pdf]

S3. Pairwise Alignment Output of LIMK1 and LIMK2 Amino Acid Sequences

EMBOSS Needle online web-tool (https://www.ebi.ac.uk/Tools/psa/emboss_needle/) was used for the alignment process. The output is provided below.

```
########################################
# Program: needle
# Rundate: Fri  1 Oct 2021 20:52:45
# Commandline: needle
#    -auto
#    -stdout
#    -asequence emboss_needle-I20211001-205244-0247-18490621-p2m.asequence
#    -bsequence emboss_needle-I20211001-205244-0247-18490621-p2m.bsequence
#    -datafile EBLOSUM62
#    -gapopen 10.0
#    -gapextend 0.5
#    -endopen 10.0
#    -endextend 0.5
#    -aformat3 pair
#    -sprotein1
#    -sprotein2
# Align_format: pair
# Report_file: stdout
########################################

#=======================================
#
# Aligned_sequences: 2
# 1: LIMK1_HUMAN
# 2: LIMK2_HUMAN
# Matrix: EBLOSUM62
# Gap_penalty: 10.0
# Extend_penalty: 0.5
#
# Length: 669
# Identity:     347/669 (51.9%)
# Similarity:   447/669 (66.8%)
# Gaps:          53/669 ( 7.9%)
# Score: 1754.5
#
#
#=======================================


LIMK1_HUMAN        1 MRLTLLCCTWREERMGEEGSELPVCASCGQRIYDGQ-YLQALNADWHADC     49
                                  .....|.::..|..||..|...| :.:.:|..||..|
LIMK2_HUMAN        1 -------------MSALAGEDVWRCPGCGDHIAPSQIWYRTVNETWHGSC     37

LIMK1_HUMAN       50 FRCCDCSASLSHQYYEKDGQLFCKKDYWARYGESCHGCSEQITKGLVMVA     99
                     |||.:|..||::.||||||:|:|.||||.::||.|||||..:| |..|||
LIMK2_HUMAN       38 FRCSECQDSLTNWYYEKDGKLYCPKDYWGKFGEFCHGCSLLMT-GPFMVA     86

LIMK1_HUMAN      100 GELKYHPECFICLTCGTFIGDGDTYTLVEHSKLYCGHCYYQTVVTPVIEQ    149
                     ||.|||||||.|::|...|.|||.|.||:|:.||||.|:.:.|:.|:.|:
LIMK2_HUMAN       87 GEFKYHPECFACMSCKVIIEDGDAYALVQHATLYCGKCHNEVVLAPMFER    136

LIMK1_HUMAN      150 ILPDSPGSHLPHTVTLVSIPASSHGKRGLSVSIDPPHGPPGCGTEHSHTV    199
                     :..:|....||::|||:|:||::.|:||.|||::     ..| :.::.||
LIMK2_HUMAN      137 LSTESVQEQLPYSVTLISMPATTEGRRGFSVSVE-----SAC-SNYATTV    180

LIMK1_HUMAN      200 RVQGVDPGCMSPDVKNSIHVGDRILEINGTPIRNVPLDEIDLLIQETSRL    249
                     :|:.|:...:||:.:|:||.|||||||||||:|.:.::|::..|.:||:.
LIMK2_HUMAN      181 QVKEVNRMHISPNNRNAIHPGDRILEINGTPVRTLRVEEVEDAISQTSQT    230

LIMK1_HUMAN      250 LQLTLEHDPHD------TLGHGLGPETSPLSSP----AYTPSGEAGSSAR    289
```

```
                     |||.:||||..      .|...|.|.......|    ..........:.|
LIMK2_HUMAN      231 LQLLIEHDPVSQRLDQLRLEARLAPHMQNAGHPHALSTLDTKENLEGTLR    280

LIMK1_HUMAN      290 QKPVLRSCSIDRSPGAGSLGSPASQRKDLGRSESLRVVCR---PHRIFRP    336
                     ::.:.||.||.:|||..|...|....:|:.||||||  |.   ..:||||
LIMK2_HUMAN      281 RRSLRRSNSISKSPGPSSPKEPLLFSRDISRSESLR--CSSSYSQQIFRP    328

LIMK1_HUMAN      337 SDLIHGEVLGKGCFGQAIKVTHRETGEVMVMKELIRFDEETQRTFLKEVK    386
                     .|||||||||||.|||||||||:.||:|||||||||.|||||:|||.|||
LIMK2_HUMAN      329 CDLIHGEVLGKGFFGQAIKVTHKATGKVMVMKELIRCDEETQKTFLTEVK    378

LIMK1_HUMAN      387 VMRCLEHPNVLKFIGVLYKDKRLNFITEYIKGGTLRGIIKSMDSQYPWSQ    436
                     |||.|:|||||||||||||||:||.:||||:||||:..::||| .:||.|
LIMK2_HUMAN      379 VMRSLDHPNVLKFIGVLYKDKKLNLLTEYIEGGTLKDFLRSMD-PFPWQQ    427

LIMK1_HUMAN      437 RVSFAKDIASGMAYLHSMNIIHRDLNSHNCLVRENKNVVVADFGLARLMV    486
                     :|.|||.|||||||||||.||||||||||||::.:|.||||||||:||:|
LIMK2_HUMAN      428 KVRFAKGIASGMAYLHSMCIIHRDLNSHNCLIKLDKTVVVADFGLSRLIV    477

LIMK1_HUMAN      487 DEKTQ------PEGLRSLKKPDRKKRYTVVGNPYWMAPEMINGRSYDEKV    530
                     :|:.:      ....|:|:|.|||||||||||||||||||:||:||||.|
LIMK2_HUMAN      478 EERKRAPMEKATTKKRTLRKNDRKKRYTVVGNPYWMAPEMLNGKSYDETV    527

LIMK1_HUMAN      531 DVFSFGIVLCEIIGRVNADPDYLPRTMDFGLNVRGFLDRYCPPNCPPSFF    580
                     |:||||||||||||:|.||||.||||:||||||:.|.:::.|.:|||:||
LIMK2_HUMAN      528 DIFSFGIVLCEIIGQVYADPDCLPRTLDFGLNVKLFWEKFVPTDCPPAFF    577

LIMK1_HUMAN      581 PITVRCCDLDPEKRPSFVKLEHWLETLRMHLAGHL--PLGPQLEQLDRGF    628
                     |:...||.|:||.||.|.|||...|.|.::| |.|  ||..:||:||...
LIMK2_HUMAN      578 PLAAICCRLEPESRPAFSKLEDSFEALSLYL-GELGIPLPAELEELDHTV    626

LIMK1_HUMAN      629 WETYRRGESGLPAHPEVPD    647
                     ...|.......|
LIMK2_HUMAN      627 SMQYGLTRDSPP-------    638


#---------------------------------------
#---------------------------------------
```
