# Supplementary material for: Protein domain-based prediction of drug/compound–target interactions and experimental validation on LIM kinases
Source: PLoS Comput Biol. 2021 Nov 29;17(11):e1009171. doi: 10.1371/journal.pcbi.1009171 (PMC8659301; doi:10.1371/journal.pcbi.1009171)

S1 Fig. Kaplan-Meier survival plots using liver cancer (HCC) RNA-seq data from 364 patients, associated with the expression profile of LIMK1 and LIMK2.

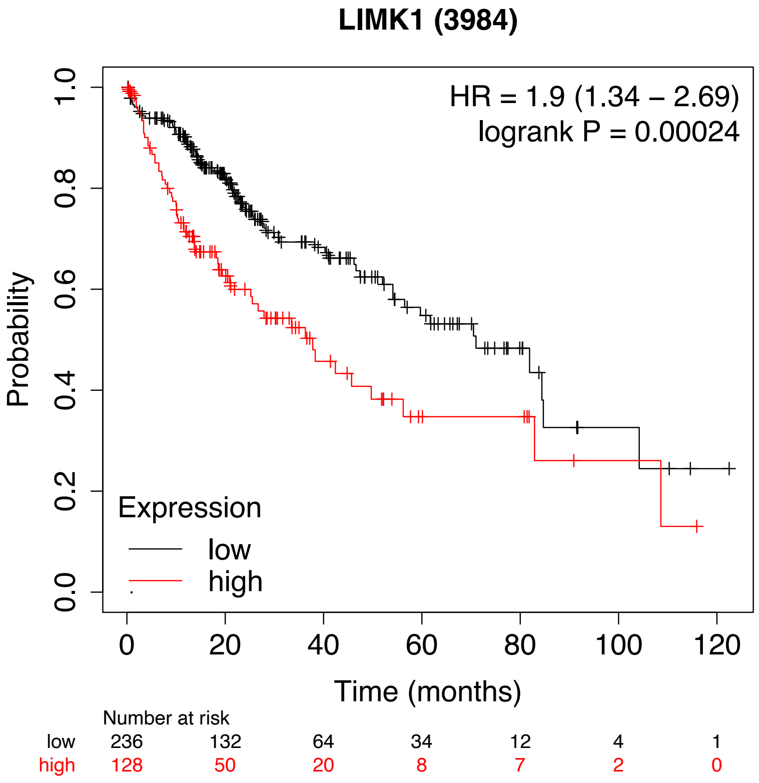

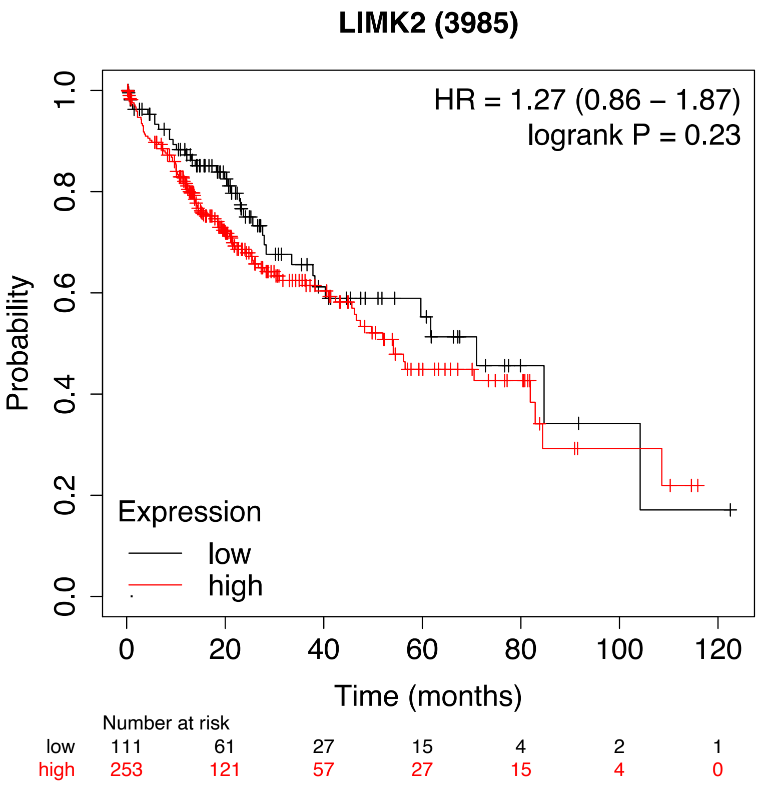

Supplement: S1 Fig — (PDF) [file pcbi.1009171.s004.pdf]
