# Supplementary material for: Protein domain-based prediction of drug/compound–target interactions and experimental validation on LIM kinases
Source: PLoS Comput Biol. 2021 Nov 29;17(11):e1009171. doi: 10.1371/journal.pcbi.1009171 (PMC8659301; doi:10.1371/journal.pcbi.1009171)
Supplement: S1 Table — (PDF) [file pcbi.1009171.s005.pdf]

S1 Table. Statistics of pathway-based compound – target protein interaction predictions.

Pathway id (KEGG)	Pathway name	# of predictions	# of compounds	# of targets
hsa00010	Glycolysis - Gluconeogenesis	48	24	9
hsa00040	Pentose and glucuronate interconversions	42	20	3
hsa00051	Fructose and mannose metabolism	22	20	2
hsa00052	Galactose metabolism	139	80	6
hsa00053	Ascorbate and aldarate metabolism	18	4	5
hsa00071	Fatty acid degradation	18	4	5
hsa00100	Steroid biosynthesis	61	61	1
hsa00120	Primary bile acid biosynthesis	41	21	2
hsa00140	Steroid hormone biosynthesis	97	21	5
hsa00190	Oxidative phosphorylation	67	67	1
hsa00220	Arginine biosynthesis	441	276	5
hsa00230	Purine metabolism	10726	2060	31
hsa00240	Pyrimidine metabolism	457	265	4
hsa00250	Alanine, aspartate and glutamate metabolism	350	254	5
hsa00260	Glycine, serine and threonine metabolism	51	30	3
hsa00270	Cysteine and methionine metabolism	55	55	2
hsa00280	Valine, leucine and isoleucine degradation	56	38	7
hsa00310	Lysine degradation	4956	604	36
hsa00330	Arginine and proline metabolism	240	98	13
hsa00340	Histidine metabolism	75	30	10
hsa00350	Tyrosine metabolism	187	134	7
hsa00360	Phenylalanine metabolism	91	38	6
hsa00380	Tryptophan metabolism	103	38	9
hsa00400	Phenylalanine, tyrosine and tryptophan biosynthesis	34	34	1
hsa00410	beta-Alanine metabolism	66	38	10
hsa00471	D-Glutamine and D-glutamate metabolism	308	216	2
hsa00480	Glutathione metabolism	21	21	1
hsa00500	Starch and sucrose metabolism	117	60	4
hsa00510	N-Glycan biosynthesis	80	80	2
hsa00513	Various types of N-glycan biosynthesis	80	80	2
hsa00515	Mannose type O-glycan biosynthesis	1878	1878	1
hsa00520	Amino sugar and nucleotide sugar metabolism	360	72	5
hsa00533	Glycosaminoglycan biosynthesis - keratan sulfate	19	19	1
hsa00561	Glycerolipid metabolism	1112	330	25
hsa00562	Inositol phosphate metabolism	1333	226	35
hsa00564	Glycerophospholipid metabolism	1251	384	24
hsa00565	Ether lipid metabolism	324	256	10
hsa00590	Arachidonic acid metabolism	550	366	15
hsa00591	Linoleic acid metabolism	277	237	7
hsa00592	alpha-Linolenic acid metabolism	268	228	6
hsa00600	Sphingolipid metabolism	20	20	2
hsa00620	Pyruvate metabolism	18	4	5
hsa00640	Propanoate metabolism	4	4	1
hsa00650	Butanoate metabolism	4	4	1
hsa00670	One carbon pool by folate	10	6	3
hsa00730	Thiamine metabolism	67	67	1
hsa00740	Riboflavin metabolism	6	6	1
hsa00760	Nicotinate and nicotinamide metabolism	257	229	9
hsa00790	Folate biosynthesis	42	23	4
hsa00830	Retinol metabolism	4	4	1
hsa00860	Porphyrin and chlorophyll metabolism	34	34	1
hsa00910	Nitrogen metabolism	3644	360	13
hsa00970	Aminoacyl-tRNA biosynthesis	15	15	1
hsa00980	Metabolism of xenobiotics by cytochrome P450	71	25	6
hsa00982	Drug metabolism - cytochrome P450	57	30	5
hsa00983	Drug metabolism - other enzymes	588	390	3
hsa01100	Metabolic pathways	25982	3748	199
hsa01200	Carbon metabolism	4	4	1
hsa01230	Biosynthesis of amino acids	4	4	1
hsa02010	ABC transporters	2829	67	44
hsa03008	Ribosome biogenesis in eukaryotes	5676	1945	6

hsa03010	Ribosome	3	1	3
hsa03013	RNA transport	143	73	11
hsa03015	mRNA surveillance pathway	231	175	10
hsa03018	RNA degradation	1958	1898	7
hsa03022	Basal transcription factors	2182	2063	5
hsa03030	DNA replication	469	67	7
hsa03040	Spliceosome	253	83	15
hsa03050	Proteasome	403	68	7
hsa03060	Protein export	134	67	2
hsa03320	PPAR signaling pathway	8066	2869	16
hsa03410	Base excision repair	56	15	4
hsa03420	Nucleotide excision repair	2211	1925	11
hsa03430	Mismatch repair	335	67	5
hsa03440	Homologous recombination	496	325	12
hsa03450	Non-homologous end-joining	8	8	2
hsa03460	Fanconi anemia pathway	347	246	11
hsa04010	MAPK signaling pathway	171532	3610	173
hsa04012	ErbB signaling pathway	69709	2184	72
hsa04014	Ras signaling pathway	98427	7829	115
hsa04015	Rap1 signaling pathway	142096	7890	115
hsa04020	Calcium signaling pathway	314419	7670	135
hsa04022	cGMP-PKG signaling pathway	121063	7872	90
hsa04024	cAMP signaling pathway	236900	8623	136
hsa04060	Cytokine-cytokine receptor interaction	132392	7348	49
hsa04061	Viral protein interaction with cytokine and cytokine receptor	104054	7153	24
hsa04062	Chemokine signaling pathway	163031	7566	92
hsa04064	NF-kappa B signaling pathway	31670	2631	42
hsa04066	HIF-1 signaling pathway	42691	2520	44
hsa04068	FoxO signaling pathway	80263	7939	67
hsa04070	Phosphatidylinositol signaling system	7701	2221	58
hsa04071	Sphingolipid signaling pathway	90891	7722	58
hsa04072	Phospholipase D signaling pathway	106418	7873	87
hsa04080	Neuroactive ligand-receptor interaction	860335	6603	242
hsa04110	Cell cycle	30236	2455	36
hsa04114	Oocyte meiosis	49036	2623	49
hsa04115	p53 signaling pathway	11371	2395	16
hsa04120	Ubiquitin mediated proteolysis	2230	2084	19
hsa04136	Autophagy - other	3659	1899	5
hsa04137	Mitophagy - animal	19792	1998	24
hsa04140	Autophagy - animal	62827	2510	56
hsa04141	Protein processing in endoplasmic reticulum	18652	2209	32
hsa04142	Lysosome	609	352	23
hsa04144	Endocytosis	58513	7788	99
hsa04145	Phagosome	3416	447	61
hsa04146	Peroxisome	719	384	10
hsa04150	mTOR signaling pathway	56847	2089	49
hsa04151	PI3K-Akt signaling pathway	149836	8849	134
hsa04152	AMPK signaling pathway	35819	8001	43
hsa04210	Apoptosis	37959	2785	72
hsa04211	Longevity regulating pathway	30645	2870	41
hsa04213	Longevity regulating pathway - multiple species	21776	2238	24
hsa04215	Apoptosis - multiple species	5605	1952	11
hsa04216	Ferroptosis	102	102	3
hsa04217	Necroptosis	27633	2305	46
hsa04218	Cellular senescence	49795	2336	80
hsa04260	Cardiac muscle contraction	460	96	13
hsa04261	Adrenergic signaling in cardiomyocytes	69904	7500	71
hsa04270	Vascular smooth muscle contraction	100852	7714	73
hsa04310	Wnt signaling pathway	59007	8609	62
hsa04330	Notch signaling pathway	2170	461	23
hsa04340	Hedgehog signaling pathway	29171	7468	25
hsa04350	TGF-beta signaling pathway	29797	2399	33
hsa04360	Axon guidance	88014	7897	134
hsa04370	VEGF signaling pathway	34847	2266	43
hsa04371	Apelin signaling pathway	53407	7957	55
hsa04380	Osteoclast differentiation	56420	2936	95

hsa04390	Hippo signaling pathway	24458	2301	47
hsa04392	Hippo signaling pathway - multiple species	9318	1989	7
hsa04510	Focal adhesion	75421	2662	111
hsa04512	ECM-receptor interaction	2779	301	38
hsa04514	Cell adhesion molecules (CAMs)	7813	496	92
hsa04520	Adherens junction	34613	2242	40
hsa04530	Tight junction	37083	2249	68
hsa04540	Gap junction	74856	7582	43
hsa04550	Signaling pathways regulating pluripotency of stem cells	52739	3149	56
hsa04610	Complement and coagulation cascades	41110	5799	49
hsa04611	Platelet activation	80685	7862	71
hsa04612	Antigen processing and presentation	3302	475	48
hsa04614	Renin-angiotensin system	21849	5478	11
hsa04620	Toll-like receptor signaling pathway	48380	2420	44
hsa04621	NOD-like receptor signaling pathway	46498	2651	75
hsa04622	RIG-I-like receptor signaling pathway	25566	2318	28
hsa04623	Cytosolic DNA-sensing pathway	11440	2127	13
hsa04625	C-type lectin receptor signaling pathway	44005	2676	77
hsa04630	JAK-STAT signaling pathway	22457	2287	51
hsa04640	Hematopoietic cell lineage	7886	1844	43
hsa04650	Natural killer cell mediated cytotoxicity	31621	2399	82
hsa04657	IL-17 signaling pathway	34722	2494	41
hsa04658	Th1 and Th2 cell differentiation	35180	2392	66
hsa04659	Th17 cell differentiation	41042	2970	68
hsa04660	T cell receptor signaling pathway	65688	2598	83
hsa04662	B cell receptor signaling pathway	29024	2591	67
hsa04664	Fc epsilon RI signaling pathway	44239	2062	48
hsa04666	Fc gamma R-mediated phagocytosis	37162	2367	58
hsa04668	TNF signaling pathway	52419	2617	71
hsa04670	Leukocyte transendothelial migration	29474	7501	55
hsa04672	Intestinal immune network for IgA production	19690	7519	27
hsa04710	Circadian rhythm	9204	2602	16
hsa04713	Circadian entrainment	38563	7512	53
hsa04714	Thermogenesis	50066	8503	45
hsa04720	Long-term potentiation	40190	2217	56
hsa04721	Synaptic vesicle cycle	535	236	17
hsa04722	Neurotrophin signaling pathway	84583	2490	86
hsa04723	Retrograde endocannabinoid signaling	33202	7442	60
hsa04724	Glutamatergic synapse	20652	2349	65
hsa04725	Cholinergic synapse	61084	7493	64
hsa04726	Serotonergic synapse	86087	8032	66
hsa04727	GABAergic synapse	14046	2399	38
hsa04728	Dopaminergic synapse	65047	7526	78
hsa04730	Long-term depression	25408	2097	36
hsa04740	Olfactory transduction	2047589	7464	405
hsa04742	Taste transduction	65428	7549	46
hsa04744	Phototransduction	13337	7466	20
hsa04750	Inflammatory mediator regulation of TRP channels	98657	7667	73
hsa04810	Regulation of actin cytoskeleton	127860	7885	99
hsa04910	Insulin signaling pathway	53995	2222	69
hsa04911	Insulin secretion	40535	7559	46
hsa04912	GnRH signaling pathway	66170	7630	68
hsa04913	Ovarian steroidogenesis	19916	7426	16
hsa04914	Progesterone-mediated oocyte maturation	56983	2598	39
hsa04915	Estrogen signaling pathway	38740	8406	70
hsa04916	Melanogenesis	41325	7538	42
hsa04917	Prolactin signaling pathway	38153	8192	49
hsa04918	Thyroid hormone synthesis	17205	7430	31
hsa04919	Thyroid hormone signaling pathway	35311	3208	61
hsa04920	Adipocytokine signaling pathway	28011	2922	32
hsa04921	Oxytocin signaling pathway	67914	7875	88
hsa04922	Glucagon signaling pathway	32279	2811	53
hsa04923	Regulation of lipolysis in adipocytes	54746	7475	28
hsa04924	Renin secretion	52592	7543	44
hsa04925	Aldosterone synthesis and secretion	47574	8263	59
hsa04926	Relaxin signaling pathway	73813	7739	69

hsa04927	Cortisol synthesis and secretion	19778	8201	38
hsa04928	Parathyroid hormone synthesis, secretion and action	30258	3318	62
hsa04929	GnRH secretion	32305	8256	45
hsa04935	Growth hormone synthesis, secretion and action	78036	7545	85
hsa04960	Aldosterone-regulated sodium reabsorption	14534	2478	17
hsa04961	Endocrine and other factor-regulated calcium reabsorption	17560	8235	20
hsa04962	Vasopressin-regulated water reabsorption	11577	7392	14
hsa04964	Proximal tubule bicarbonate reclamation	791	528	4
hsa04966	Collecting duct acid secretion	183	183	1
hsa04970	Salivary secretion	51624	7595	34
hsa04971	Gastric acid secretion	46915	7672	37
hsa04972	Pancreatic secretion	17810	7806	33
hsa04973	Carbohydrate digestion and absorption	7374	1999	22
hsa04974	Protein digestion and absorption	1487	380	21
hsa04975	Fat digestion and absorption	308	109	7
hsa04976	Bile secretion	8273	2922	27
hsa04977	Vitamin digestion and absorption	235	206	6
hsa04978	Mineral absorption	901	781	6
hsa04979	Cholesterol metabolism	703	212	14
