# Supplementary material for: Protein domain-based prediction of drug/compound–target interactions and experimental validation on LIM kinases
Source: PLoS Comput Biol. 2021 Nov 29;17(11):e1009171. doi: 10.1371/journal.pcbi.1009171 (PMC8659301; doi:10.1371/journal.pcbi.1009171)
Supplement: S2 Table — (PDF) [file pcbi.1009171.s006.pdf]

S2 Table. Compound – target pairs that are reported to have a bioactivity value between 10 and 20 μM in ChEMBL (v29), and predicted as active/interacting by DRUIDom.

**Compound_ChEMBL_id – Target_UniProt_acc**				
CHEMBL105608 – Q6W5P4	CHEMBL1397874 – Q9H228	CHEMBL1922120 – P06493	CHEMBL286721 – P49759	CHEMBL490935 – P29474
CHEMBL1081382 – P27338	CHEMBL1401508 – Q16790	CHEMBL1922209 – P06493	CHEMBL288174 – P16473	CHEMBL500996 – Q6W5P4
CHEMBL1088158 – O43451	CHEMBL1401508 – Q9ULX7	CHEMBL1922209 – P11802	CHEMBL288174 – Q6W5P4	CHEMBL55267 – Q05655
CHEMBL1095574 – P22748	CHEMBL1404207 – Q96LD8	CHEMBL1922209 – P28482	CHEMBL293720 – P22303	CHEMBL553 – P04049
CHEMBL111 – Q14330	CHEMBL1404207 – Q9BQF6	CHEMBL1922212 – P11802	CHEMBL296103 – Q02763	CHEMBL553 – P09619
CHEMBL1112 – Q6W5P4	CHEMBL1404207 – Q9GZR1	CHEMBL1922218 – P11802	CHEMBL296419 – P08588	CHEMBL56543 – P04626
CHEMBL1113 – P16473	CHEMBL1408579 – P54829	CHEMBL1923968 – P07384	CHEMBL296419 – P32245	CHEMBL567341 – P07858
CHEMBL1162193 – P41231	CHEMBL1410669 – P16473	CHEMBL1929302 – P00533	CHEMBL296419 – P41968	CHEMBL568173 – P43166
CHEMBL1172 – Q9H3N8	CHEMBL1414 – P22748	CHEMBL194805 – Q92731	CHEMBL296455 – P28482	CHEMBL568348 – P07451
CHEMBL117697 – P29376	CHEMBL1415555 – O95136	CHEMBL195437 – P29274	CHEMBL298612 – P28335	CHEMBL568416 – P00918
CHEMBL117697 – P35968	CHEMBL1417177 – Q9HBX9	CHEMBL19612 – P07451	CHEMBL298612 – P35368	CHEMBL569185 – Q12809
CHEMBL117697 – Q07912	CHEMBL1421770 – P41143	CHEMBL198039 – P50281	CHEMBL300987 – P20309	CHEMBL570360 – Q12809
CHEMBL117697 – Q96GD4	CHEMBL1431953 – P21554	CHEMBL1981675 – P28482	CHEMBL312862 – O75469	CHEMBL570805 – Q12809
CHEMBL1182210 – P16473	CHEMBL1433815 – P16473	CHEMBL1981770 – P28482	CHEMBL321510 – P00749	CHEMBL571952 – Q12809
CHEMBL1186802 – P35228	CHEMBL1434729 – P16473	CHEMBL198262 – P50281	CHEMBL321510 – P07477	CHEMBL572153 – Q12809
CHEMBL11909 – Q9HBX9	CHEMBL1446078 – Q6W5P4	CHEMBL1988859 – P28482	CHEMBL3216112 – P29474	CHEMBL578061 – O14757
CHEMBL119709 – Q9HBX9	CHEMBL1453928 – P16473	CHEMBL201511 – P06213	CHEMBL326450 – P08912	CHEMBL578061 – P05129
CHEMBL1200503 – Q6W5P4	CHEMBL1472131 – P28482	CHEMBL204077 – P00915	CHEMBL326450 – P11229	CHEMBL578061 – P11309
CHEMBL1200916 – Q6W5P4	CHEMBL1473849 – P16473	CHEMBL2062335 – P49146	CHEMBL328907 – P00519	CHEMBL578061 – P23443
CHEMBL1201049 – Q6W5P4	CHEMBL1474532 – P16473	CHEMBL2153621 – P29274	CHEMBL329033 – P06239	CHEMBL578061 – P68400
CHEMBL1201102 – Q6W5P4	CHEMBL14762 – P28482	CHEMBL2153626 – P30542	CHEMBL335065 – P14151	CHEMBL578061 – Q05655
CHEMBL1214248 – O43570	CHEMBL1485765 – P16473	CHEMBL2153627 – P29275	CHEMBL335065 – P16109	CHEMBL58228 – P11229
CHEMBL1214248 – P00918	CHEMBL1491258 – Q6W5P4	CHEMBL21731 – P16473	CHEMBL3355482 – P06239	CHEMBL583430 – Q12809
CHEMBL1214248 – P07451	CHEMBL1503193 – P16473	CHEMBL2177587 – P56524	CHEMBL344104 – P00742	CHEMBL585163 – Q12809
CHEMBL1214248 – Q16790	CHEMBL1521291 – P30556	CHEMBL2177587 – Q96DB2	CHEMBL356623 – P28221	CHEMBL590047 – Q02156
CHEMBL1230640 – Q9Y2K7	CHEMBL1525753 – P10828	CHEMBL2177587 – Q9UKV0	CHEMBL3621860 – Q9H3R0	CHEMBL593165 – Q16790
CHEMBL1232474 – P16473	CHEMBL1534846 – Q9HBX9	CHEMBL2177588 – Q9UKV0	CHEMBL3621868 – Q9H3R0	CHEMBL593618 – Q16790
CHEMBL1233715 – P35228	CHEMBL1542448 – P34972	CHEMBL2203713 – P18825	CHEMBL3621871 – Q9Y2K7	CHEMBL59939 – P08172
CHEMBL1241680 – P54760	CHEMBL1562420 – P28482	CHEMBL2303665 – P16581	CHEMBL3621875 – Q9Y2K7	CHEMBL606245 – P07948
CHEMBL1241771 – P54760	CHEMBL1570251 – P16473	CHEMBL2316903 – P08575	CHEMBL3621876 – Q9H3R0	CHEMBL606245 – P24941
CHEMBL1242208 – P35968	CHEMBL1581985 – P16473	CHEMBL2316903 – P17706	CHEMBL3621878 – Q9H3R0	CHEMBL606245 – Q15418
CHEMBL1242208 – P54760	CHEMBL1583874 – P28482	CHEMBL2316903 – P18031	CHEMBL3621879 – Q9H3R0	CHEMBL612083 – P16473
CHEMBL1242472 – P35968	CHEMBL1583874 – P53779	CHEMBL2316903 – P43378	CHEMBL3621881 – O75164	CHEMBL6291 – Q86Y07
CHEMBL1256656 – Q6W5P4	CHEMBL1592119 – Q9H228	CHEMBL2316904 – P18031	CHEMBL3621881 – Q9H3R0	CHEMBL63329 – Q6W5P4
CHEMBL1256761 – Q6W5P4	CHEMBL1592223 – P24941	CHEMBL23194 – Q16790	CHEMBL363807 – P29275	CHEMBL656 – P41145
CHEMBL1256958 – Q9HBX9	CHEMBL1613623 – P16473	CHEMBL2324339 – P51878	CHEMBL385168 – P41143	CHEMBL69863 – Q15746
CHEMBL1277786 – P35228	CHEMBL1659 – Q6W5P4	CHEMBL2326408 – P10828	CHEMBL388239 – P03952	CHEMBL71 – P32245
CHEMBL127907 – P11362	CHEMBL168829 – P00533	CHEMBL2326408 – P11474	CHEMBL388978 – Q9H4A3	CHEMBL71851 – P00915
CHEMBL1294 – Q13936	CHEMBL1702752 – P35372	CHEMBL2373635 – Q6W5P4	CHEMBL403128 – P28482	CHEMBL72365 – P28482
CHEMBL130266 – P41231	CHEMBL1704084 – P34972	CHEMBL2374027 – Q6W5P4	CHEMBL404941 – P48729	CHEMBL752 – Q96G91
CHEMBL130266 – P51582	CHEMBL1705 – Q6W5P4	CHEMBL2374078 – Q6W5P4	CHEMBL406266 – Q15077	CHEMBL77966 – P08183
CHEMBL1307207 – Q9BQF6	CHEMBL1707517 – Q9HC97	CHEMBL239233 – P0DMS8	CHEMBL40966 – P13945	CHEMBL7917 – P28482
CHEMBL1311617 – P16473	CHEMBL1710 – Q6W5P4	CHEMBL2418740 – P35372	CHEMBL41632 – P19793	CHEMBL7917 – Q99640
CHEMBL1314751 – Q6W5P4	CHEMBL1711929 – P35372	CHEMBL2418740 – P41143	CHEMBL425020 – P50281	CHEMBL80895 – P28482
CHEMBL1317978 – P16473	CHEMBL1713 – Q6W5P4	CHEMBL2418741 – P35372	CHEMBL427366 – P50281	CHEMBL87563 – P00750
CHEMBL1319733 – P10828	CHEMBL1713943 – P35372	CHEMBL245968 – Q02763	CHEMBL43064 – Q6W5P4	CHEMBL92086 – P04626
CHEMBL1322702 – Q6W5P4	CHEMBL1715925 – Q96EB6	CHEMBL246356 – P10721	CHEMBL431276 – P35372	CHEMBL92251 – P00734
CHEMBL1327758 – P16473	CHEMBL1715925 – Q9NXA8	CHEMBL25236 – P16473	CHEMBL431342 – Q02763	
CHEMBL1336 – Q9Y572	CHEMBL1736339 – P35372	CHEMBL253551 – O43570	CHEMBL441343 – P00918	
CHEMBL1357429 – Q13627	CHEMBL178 – P10828	CHEMBL25610 – P11362	CHEMBL450234 – P29475	
CHEMBL1358012 – P16473	CHEMBL1783963 – P50406	CHEMBL258161 – P08912	CHEMBL459729 – P17252	
CHEMBL1359181 – Q6W5P4	CHEMBL1784886 – Q15077	CHEMBL258805 – P09619	CHEMBL466841 – P00918	
CHEMBL137780 – P00749	CHEMBL185327 – P00533	CHEMBL258893 – P28482	CHEMBL476833 – Q9Y2T6	
CHEMBL1397874 – Q99500	CHEMBL189584 – P41240	CHEMBL26998 – P16473	CHEMBL478629 – P19525	
