# Supplementary material for: Protein domain-based prediction of drug/compound–target interactions and experimental validation on LIM kinases
Source: PLoS Comput Biol. 2021 Nov 29;17(11):e1009171. doi: 10.1371/journal.pcbi.1009171 (PMC8659301; doi:10.1371/journal.pcbi.1009171)
Supplement: S3 Table — (PDF) [file pcbi.1009171.s007.pdf]

**S3 Table.** Bioactive compound predictions for PI3K/AKT/mTOR pathway proteins: LIMK1, LIMK2, MDM2 and VEGFA; given as ChEMBL molecule identifiers and gene names of the corresponding targets.

| **Predicted Compound (ChEMBL id)** | **Target Protein (Gene Name)** | **Predicted Compound (ChEMBL id)** | **Target Protein (Gene Name)** |
|---|---|---|---|
| CHEMBL1316589 | LIMK1 | CHEMBL505899 | MDM2 |
| CHEMBL1512352 | LIMK1 | CHEMBL506261 | MDM2 |
| CHEMBL516650 | LIMK1 | CHEMBL506263 | MDM2 |
| CHEMBL518653 | LIMK1 | CHEMBL506507 | MDM2 |
| CHEMBL1316589 | LIMK2 | CHEMBL506623 | MDM2 |
| CHEMBL1512352 | LIMK2 | CHEMBL506646 | MDM2 |
| CHEMBL516650 | LIMK2 | CHEMBL506647 | MDM2 |
| CHEMBL518653 | LIMK2 | CHEMBL506740 | MDM2 |
| CHEMBL1241424 | MDM2 | CHEMBL507004 | MDM2 |
| CHEMBL1241425 | MDM2 | CHEMBL507649 | MDM2 |
| CHEMBL1241426 | MDM2 | CHEMBL508126 | MDM2 |
| CHEMBL1243385 | MDM2 | CHEMBL508377 | MDM2 |
| CHEMBL1242922 | MDM2 | CHEMBL508398 | MDM2 |
| CHEMBL458791 | MDM2 | CHEMBL508486 | MDM2 |
| CHEMBL514738 | MDM2 | CHEMBL508491 | MDM2 |
| CHEMBL515347 | MDM2 | CHEMBL508564 | MDM2 |
| CHEMBL515848 | MDM2 | CHEMBL508902 | MDM2 |
| CHEMBL516172 | MDM2 | CHEMBL508983 | MDM2 |
| CHEMBL475670 | MDM2 | CHEMBL509409 | MDM2 |
| CHEMBL481213 | MDM2 | CHEMBL509666 | MDM2 |
| CHEMBL481421 | MDM2 | CHEMBL510017 | MDM2 |
| CHEMBL1791379 | MDM2 | CHEMBL510066 | MDM2 |
| CHEMBL1791380 | MDM2 | CHEMBL510233 | MDM2 |
| CHEMBL1791382 | MDM2 | CHEMBL510473 | MDM2 |
| CHEMBL219860 | MDM2 | CHEMBL510817 | MDM2 |
| CHEMBL434556 | MDM2 | CHEMBL511030 | MDM2 |
| CHEMBL427239 | MDM2 | CHEMBL524509 | MDM2 |
| CHEMBL1791381 | MDM2 | CHEMBL524659 | MDM2 |
| CHEMBL445253 | MDM2 | CHEMBL524691 | MDM2 |
| CHEMBL505051 | MDM2 | CHEMBL524856 | MDM2 |
| CHEMBL503520 | MDM2 | CHEMBL524887 | MDM2 |
| CHEMBL207341 | MDM2 | CHEMBL524908 | MDM2 |
| CHEMBL443697 | MDM2 | CHEMBL525014 | MDM2 |
| CHEMBL446284 | MDM2 | CHEMBL525018 | MDM2 |
| CHEMBL450322 | MDM2 | CHEMBL525040 | MDM2 |
| CHEMBL451424 | MDM2 | CHEMBL525045 | MDM2 |
| CHEMBL451944 | MDM2 | CHEMBL525060 | MDM2 |

| | | | |
|---|---|---|---|
| CHEMBL454229 | MDM2 | CHEMBL525201 | MDM2 |
| CHEMBL486090 | MDM2 | CHEMBL525263 | MDM2 |
| CHEMBL499121 | MDM2 | CHEMBL525265 | MDM2 |
| CHEMBL499749 | MDM2 | CHEMBL525594 | MDM2 |
| CHEMBL499766 | MDM2 | CHEMBL525614 | MDM2 |
| CHEMBL500441 | MDM2 | CHEMBL525624 | MDM2 |
| CHEMBL500788 | MDM2 | CHEMBL525635 | MDM2 |
| CHEMBL501541 | MDM2 | CHEMBL525636 | MDM2 |
| CHEMBL503191 | MDM2 | CHEMBL526187 | MDM2 |
| CHEMBL503489 | MDM2 | CHEMBL526336 | MDM2 |
| CHEMBL503730 | MDM2 | CHEMBL526337 | MDM2 |
| CHEMBL503983 | MDM2 | CHEMBL526381 | MDM2 |
| CHEMBL504226 | MDM2 | CHEMBL526861 | MDM2 |
| CHEMBL504266 | MDM2 | CHEMBL527080 | MDM2 |
| CHEMBL504423 | MDM2 | CHEMBL527084 | MDM2 |
| CHEMBL504493 | MDM2 | CHEMBL1089944 | VEGFA |
| CHEMBL504855 | MDM2 | CHEMBL1689394 | VEGFA |
| CHEMBL504919 | MDM2 | CHEMBL499790 | VEGFA |
| CHEMBL505501 | MDM2 | CHEMBL501558 | VEGFA |
| CHEMBL505622 | MDM2 | CHEMBL508411 | VEGFA |
| CHEMBL505790 | MDM2 | CHEMBL509774 | VEGFA |
