# Supplementary material for: Protein domain-based prediction of drug/compound–target interactions and experimental validation on LIM kinases
Source: PLoS Comput Biol. 2021 Nov 29;17(11):e1009171. doi: 10.1371/journal.pcbi.1009171 (PMC8659301; doi:10.1371/journal.pcbi.1009171)
Supplement: S4 Table — (PDF) [file pcbi.1009171.s008.pdf]

S4 Table. Full-scale molecular docking results (including both local and online docking) of computationally predicted LIMK inhibitors and their derivatives against kinase domains of LIMK proteins in terms of binding free energy (ΔG).

LIMK1	Molecule file resource	Local Molecular Docking Results		Online Molecular Docking Results	
		AutoDock (Rigid)	AutoDock (Flexible)	MtiAutoDock	SwissDock
**Native ligand (Staurosporine)**	ZINC	-9.5	-10.6	-11.8	-9.9
**LİMKİ-2 (M-379)**	ZINC	-9.1	-10.1	-9.8	-8.4
**LİMKİ-3 (M-395)**	ZINC	-9.9	-10.0	-10.1	-8.5
**Native ligand (Staurosporine)**	Chemaxon	-9.8	-9.5	-8.9	-8.7
**LIMKi-1**	Chemaxon	-7.5	-7.7	-7.2	-7.9
**LIMKi-1a**	Chemaxon	-7.5	-7.4	-7.4	-7.8
**LIMKi-2 (M-379)**	Chemaxon	-8.4	-10.1	-8.4	-8.6
**LIMKi-2a**	Chemaxon	-8.8	-9.7	-8.9	-8.4
**LIMKi-2b**	Chemaxon	-8.0	-9.1	-8.3	-8.5
**LIMKi-2c**	Chemaxon	-9.0	-9.7	-9.8	-8.8
**LIMKi-2d**	Chemaxon	-9.5	-10.3	-9.4	-8.6
**LIMKi-3 (M-395)**	Chemaxon	-8.4	-9.3	-8.4	-8.6

LIMK2	Molecule file resource	Local Molecular Docking Results		Online Molecular Docking Results	
		AutoDock (Rigid)	AutoDock (Flexible)	MtiAutoDock	SwissDock
**Native ligand (9D8)**	ZINC	-12.4	-9.9	-15.0	-10.7
**LİMKİ2 (M-379) - ZINC**	ZINC	-12.1	-11.4	-12.7	-9.6
**LİMKİ3 (M-395) - ZINC**	ZINC	-11.9	-11.8	-13.1	-9.4
**Native ligand (9D8)**	Chemaxon	-12.4	-12.7	-12.0	-11.5
**LIMKi-1**	Chemaxon	-9.3	-9.9	-9.3	-8.8
**LIMKi-1a**	Chemaxon	-9.3	-9.0	-9.4	-8.6
**LIMKi-2 (M-379)**	Chemaxon	-10.8	-11.8	-11.7	-9.5
**LIMKi-2a**	Chemaxon	-11.1	-11.3	-11.1	-9.5
**LIMKi-2b**	Chemaxon	-11.0	-10.5	-11.3	-9.5
**LIMKi-2c**	Chemaxon	-11.4	-11.9	-11.6	-9.7
**LIMKi-2d**	Chemaxon	-11.6	-12.0	-11.6	-9.7
**LIMKi-3 (M-395)**	Chemaxon	-10.6	-10.5	-11.4	-10.5
