# Supplementary material for: Protein domain-based prediction of drug/compound–target interactions and experimental validation on LIM kinases
Source: PLoS Comput Biol. 2021 Nov 29;17(11):e1009171. doi: 10.1371/journal.pcbi.1009171 (PMC8659301; doi:10.1371/journal.pcbi.1009171)
Supplement: Attachment — Submitted filename: DRUIDom_Manuscript_PLOSCB_Response_Letter.pdf [file pcbi.1009171.s010.pdf]

Response to Reviewers (PCOMPBIOL-D-21-01053)

We would like to thank our editor and reviewers for their in-depth evaluation, constructive comments and suggestions. To address the reviewer's comments, we carried out a comprehensive revision. We performed new analyses and improved the existing ones and revised the text accordingly. We think we addressed all of the issues raised by our reviewers, and that our manuscript has been improved significantly with revision we carried out based on this valuable feedback. Detailed point by point descriptions of our modifications in response to the specific comments raised by our reviewers, and the changes we have made in the manuscript, are given below. Reviewer comments are in bold font, our responses are provided in normal font, and manuscript modifications are in italic.

Sincerely,

Tunca Dogan, PhD
Rengul Cetin-Atalay, MD PhD
(On behalf of all authors of the manuscript)

Reviewer #1:

Please address the followed questions:

1. How can you guarantee the accuracy of the predicted complex structures of ligand-protein? In other word, how do you choose the predicted complex structures among the various candidate poses given by docking simulations.

As far as we are aware, it is not possible to fully guarantee that any selected docking pose would correspond to the native complex, without further validation in terms of experimental structural characterization (e.g., Xray crystallization). Since these experimental approaches are extremely expensive and time consuming, they are rarely used for the validation of computational predictions. In this study, we were able to perform validation experiments via two independent techniques: 1) in vitro experimental cell-based assays, and 2) independent computational experiments via molecular docking.

While selecting the pose to report among many docking poses, we used the one with the lowest binding free energy, as this is the most widely used approach in the literature. However, in order to prevent bias in reporting as much as possible, we repeated these docking experiments with different tools and parameters and

reported their full results in the supplementary material. The pose with lowest energy is the main reported result of the automated docking tools in this study (i.e., ranked as the first one in the results list).

Here, we also reported docking results of the native ligands of both LIMK1 and LIMK2 (i.e., staurosporine and 9D8, respectively), which are experimentally known to bind to corresponding receptors with high affinity, and docking results of the predicted compounds should be evaluated in comparison to the results of native ligands. Predicted ligands which have binding free energies that are in the same scale of the native ligands can be considered to have similar affinities.

We addressed this comment in the section: "2.4.2 Molecular Docking of Novel LIMK Inhibitors" of our manuscript, with the following text. In addition, we provided docking results from other tools/parameters in the revised version of supplementary material (Table S1).

"*... Multi-kinase inhibitor staurosporine and previously described LIMK inhibitor 9D8 have published crystal structures with the kinase domains of LIMK1 and LIMK2 proteins. These molecules were used as reference for docking, i.e., docking-based binding free energy (ΔG) output of computationally predicted LIMK inhibitors are evaluated in comparison to the docking output of these native ligands. In addition to computationally predicted compounds (i.e., LIMKi-1, LIMKi-1a, LIMKi-2 and LIMKi-3), novel derivatives of LIMKi-2 (i.e., LIMKi-2a, LIMKi-2b, LIMKi-2c and LIMKi-2d) were also docked against kinase domains of LIMK1 and LIMK2 proteins. AutoDock grid box parameters used in these analyses are displayed in Table 2a, and the docking results of each LIMK protein – compound combination are shown in Table 2b, which displays the lowest of the binding free energies calculated from several poses obtained either from rigid or flexible docking in AutoDock. All files and results of the docking analysis, including the ones for online MTiAutoDock and SwissDock docking runs, are available in the data repository of the study (https://github.com/cansyl/DRUIDom) and in Table S4, respectively. Docking results obtained from different tools are consistent with each other. ...*"

2. For some compound-target bioactivity data, different labs may give different values, how did you treat such kind of data? and why?

To be used in the training of our system (or any statistical/machine learning model) a sample should have a single and definitive label. Following our filtering data operations (which are explained in sub-section: "4.1 Dataset Construction"), if there are still multiple reported bioactivity values between a particular compound and target, we take the median activity value into account. As far as we are aware, this is

one of the simplest and realistic solutions to address the requirement to reduce multiple values into one. This solution has also been used in our previous studies as well (Ref:1).

To address this comment, we added the following text into sub-section: “4.1 Dataset Construction”, and explained how the data is treated:

“*… Following these filtering operations, there were still cases where multiple bioactivity values are reported between a particular compound and target (i.e., duplicates). We thus take the median bioactivity value into account to treat these cases, similar to previous studies [10]*”

(Ref:1) Rifaioglu, A.S., Nalbat, E., Atalay, M.V., Martin, M.J., Cetin-Atalay, R. & Doğan, T. (2020). DEEPScreen: High Performance Drug-Target Interaction Prediction with Convolutional Neural Networks Using 2-D Structural Compound Representations. *Chemical Science, 11*(9), 2531-2557.

3. It is interesting to see how does your trained model give the evaluation of the compound-target protein relations with bioactivity levels of xC50 values between 10uM~20uM?

The actual reason behind leaving bioactivity data points with values between 10 and 20 uM was that their binary activity label (i.e., as active or inactive) is ambiguous, since bioactivities in this range are neither reported to be active nor inactive in the literature, lying somewhere in the so-called twilight zone (at least for the members of many target protein families).

To address this comment, we added the following analysis and text to the manuscript at the end of the section “2.3 Predicting New Drug/Compound – Target Protein Interactions”:

“*… As explained in the dataset construction section (4.1), we discarded bioactivity data points between 10 and 20 μM from our training dataset as these are neither considered to be active nor inactive with any certainty. Thus, with the aim of observing how many of the compound – target pairs that fall into this bioactivity range (in the current version -v29- of ChEMBL) are predicted to be active/interacting by DRUIDom, we searched for these pairs among our finalized compound – target interaction predictions. Out of the 178,089 unique compound – target pairs with reported bioactivities between 10 and 20 μM, 263 of them are predicted to be active/interacting by our method (i.e., 0.15% of them), which are given in Table S2. This low number can be attributed to the fact that, most of the compounds in bioactivity databases are presented in only one (or just a few) compound – target*

pairs, and since pairs that fall into the 10-to-20 μM range have been omitted from the training dataset of DRUIDom in the first place, their compounds are missing from predictions (if they are not re-included due to ligand similarity-based extension of mappings). This is supported by the following statistics; among these 178,089 pairs, there are 124,189 unique compounds, and only 1483 of them are presented in all DRUIDom predictions. It is also important to state that this is a favorable finding, since it is not desirable to produce active/interacting predictions to pairs with ambiguous relationships in reality. In the dataset of compound – target pairs with 10-to-20 μM bioactivities, pairs that are predicted to be active by DRUIDom have a slightly lower median bioactivity value (14.1 μM), i.e., more bioactive with lower xC50 values, compared to pairs that are not predicted to be active (mean bioactivity: 14.7 μM)."

4. The resolution of the figures is too low to read.

To address this issue, we now provided the original high-quality figures as individual files. We believe that the quality is acceptable with these newly submitted files.

Reviewer #2:

In this paper, the authors use the idea of protein domains and compound similarity to predict drug-target interactions. In particular, they use non-structural associations between proteins and drugs to infer domain-drug correlations and then validate the inferred relationships on a structurally derived dataset of domain-small molecule interactions, Interacdome. Next they assume that similar drugs would bind the same domains, and ultimately transfer information about drug-domain interactions to protein sequences. They experimentally test some predictions as well.

1. Overall, this work presents interesting ideas that I haven't quite seen in the literature, and thus is of value. However, the paper is pretty verbose in parts and it's hard to figure out what exactly was done. A self contained, crisp methods section would help a lot. I think a specific method for associating drugs and domains -- ie independent of validation -- would be beneficial.

To address this comment, we modified the Methods section by relocating sub-sections related to the *in vitro* experiments to the supplementary material document. We also made additional minor changes to increase the readability (e.g., adding a short paragraph at the beginning of the Methods section to explain the sub-sections,

and including a few sub-section titles). Please refer to the Methods section in the revised manuscript for the changes.

2. The authors may find some of the statistical ideas in methods to infer domain-domain interactions from protein-protein interactions useful. Using such an approach, they could then more rigorously test their predictions on interacdome (with the caveat that this is likely to be an incomplete "gold standard").

With the aim of addressing this comment, we reviewed domain-domain interaction (DDI) prediction literature and investigated various studies in this field. Among these, a few studies came forward and highly influenced later works (Ref:1,2).

In many of these papers, DDI prediction performance was evaluated by first translating these DDI predictions to protein-protein interaction (PPI) predictions (by assuming that two proteins containing interacting domains will interact with each other), and comparing the predicted PPIs with known/true PPIs (in terms of binary classification as interaction exists or not) using various metrics, some of which we have already integrated in our study. The reason behind this indirect evaluation is that, there is no reliable reference/gold-standard resource for DDIs.

In our study, compound-domain mapping performance was calculated based on the dataset of InteracDome, which also is a compound-domain association-based method. As a result, we incorporated a new metric (i.e., enrichment over random associations of the dataset with the same size, calculated at each mapping threshold score), inspired from abovementioned DDI prediction studies (Ref:1,2), to measure the performance, once again, based on the InteracDome dataset. With the aim of addressing this issue, we have added the following text to section: “2.1 Predictive Performance Analysis”:

“*… Here, InteracDome represents an incomplete reference dataset, as a result, DRUIDom’s compound – domain mappings which are not presented in the InteracDome dataset are not necessarily false positives. In cases like this, it is important to focus on performance in terms of detecting known/true (positive) associations. Inspired from a domain-domain interaction prediction study [25, 26], we calculated the enrichment of reference compound – domain associations in highest-ranking DRUIDom mappings (accompanied with its statistical significance value) with respect to random mappings on the exact same domain and compound datasets. For this, we ranked DRUIDom mappings according to pre-calculated MCC-based mapping scores. At each arbitrarily selected mapping score threshold, we calculated the number of InteracDome associations found in DRUIDom mappings, in*

comparison to the expected number of InteracDome associations to be retrieved by randomly selecting the same number of pairs. For calculating the enrichment scores and their statistical significance, we used the hypergeometric test, as described in section "4.3 Mapping Score and Performance Analysis Metrics" equations 6 and 7. According to results, the highest enrichment scores (i.e., > 6) are achieved when the mapping score threshold is selected between 0.1 and 0.6, all of which are found to be statistically significant with p-values < 10^{-16} (Table 1). It is also indicated from enrichment results that, at very low and high mapping thresholds enrichment is low, thus, selecting these points may not be ideal. …"

(Ref:1) Riley, R., Lee, C., Sabatti, C., & Eisenberg, D. (2005). Inferring protein domain interactions from databases of interacting proteins. Genome biology, 6(10), 1-17.

(Ref:1) Lee, H., Deng, M., Sun, F., & Chen, T. (2006). An integrated approach to the prediction of domain-domain interactions. BMC bioinformatics, 7(1), 1-15.

Reviewer #3:

1. The assumption behind the mapping between domains and compounds is that either the binding region of the ligand is on the mapped structural domain(s), or there is a functional relationship between the two so that the mapped domain is required for the corresponding bioactivity to occur. (page 7) --> It is not clearly explained how functional relationships can make mapping between domains and compounds. What's the meaning in terms of the biochemistry view?

We thank our reviewer and appreciate her/his comment. What we meant here actually is the indirect/long-distance allosteric interactions. It is possible to detect cases where an allosteric site resides within a domain, as a high scoring compound – domain pair in our mappings (since there is no binding site/region information in large-scale bioactivity databases which are used as our resource). As a result, we preferred to express this possibility, even though it has an extremely low probability of occurrence compared to the cases involving direct binding. We understand that the term we used in this sense was confusing especially due to the fact that this term is used for the type of relationships such as agonism, antagonism, etc. To address this issue, we removed all of the uses of the term: from the manuscript because it would be a somehow groundless claim without a thorough analysis including example cases.

2. It is not clear on the explanations on coverage extensions on page 11.

With the aim of addressing this issue, we have added the following text to the relevant part of the manuscript (under section 2.1):

"… We also calculated coverage extension values at each mapping score threshold to indicate the numbers of new domains and compounds that have been included in DRUIDom mappings (which are not presented in the InteracDome mappings at all). Coverage extensions are given in terms of rates (percentages) calculated with respect to the total number of domains (i.e., 1,043) and compounds (i.e., 1,144) intersecting between DRUIDom and InteracDome mappings (e.g., in the case of score threshold: 0.5, DRUIDom has mappings for 250 unique domains, 50 of which is shared with InteracDome, and it has been previously calculated that the total number of intersecting domains between DRUIDom and InteracDome is 1,043, as a result, the domain coverage extension at this point is calculated as: (250-50)/1043 = 0.192, in other words: 19.2%). Considering coverage extension values, our mappings enriched the InteracDome dataset by ~19% for domains and ~707% for compounds (at the selected score threshold of 0.5), which indicate the added value of our approach, especially in terms of mapping new ligands. …"

3. The Authors should provide statistics on the number of proteins/findings of all pathways not only the PI3K pathway for information to the Readers.

To address this comment, we prepared 2 new tables: 1) a compound-target predictions list where the name of the pathway, in which the corresponding target protein take part, is given in an additional column; and 2) a statistics table which contains the number of new ligand predictions and the number of member proteins with at least one new ligand prediction, for each signalling pathway. We referenced these tables in section "2.3 Predicting New Drug/Compound – Target Protein Interactions" with the text given below:

"*… With the aim of making this long list of predictions more accessible, we grouped them based on signaling and metabolic pathways, in which the corresponding target proteins take roles. We believe this pathway-based classification will be useful to researchers, especially for the cases where specific biological processes are to be targeted (rather than a predefined single protein). Out of 5,050,841 interaction predictions, 3,686,558 of them contain target proteins that are annotated to one or more than one of 212 KEGG [36] signaling and metabolic pathways. We extracted pathway-based prediction statistics, including the number of interactions, and the*

number of unique compounds and proteins, for each pathway (Table S1), which showed that olfactory transduction, neuroactive ligand-receptor interaction and calcium signaling pathways have the highest number of predicted ligand interactions. These predictions can be potentially used in future drug discovery/repurposing studies. Both the whole and pathway-based grouped compound – target interaction prediction lists are made available in the GitHub repository of the study (https://github.com/cansyl/DRUIDom). …"

4. It needs more explanation why the Author focused on LIMK among four PI3K pathway proteins.

To address this issue, we added the following information at the beginning of the section: "2.4 Validation of Predicted Molecular Interactions" by writing a new sub-section ("2.4.1 Selection of Target Proteins"):

"*For in vitro and in silico experimental validation, we focused on the hepatocellular carcinoma (HCC) disease (i.e., a sub-type of liver cancer), which is estimated to be the fourth most deadly cancer type [37], and the PI3K/AKT/mTOR signalling pathway, mainly due to the critical role of this pathway in various types of cancer [29]. To select inhibitory compound predictions, we first checked our large-scale drug/compound – target interaction prediction dataset and found 116 inhibitor predictions (Table 2) for 4 PI3K/AKT/mTOR signalling pathway genes/proteins (i.e., VEGFA, MDM2, LIMK1 and LIMK2). Out of these 4 genes, VEGFA and MDM2 are relatively well studied effectors of liver cancer, and there are several drugs and drug candidates that are being studied in the context of targeting these proteins as reported in the literature (please see https://www.ebi.ac.uk/chembl/target_report_card/CHEMBL1783 and https://www.ebi.ac.uk/chembl/target_report_card/CHEMBL5023, for VEGFA and MDM2, respectively). However, it is not possible to state the same for LIM kinases.*

The invasiveness of cancer cells is based on changes in control mechanisms that regulate cytoskeletal remodeling and cell migration. LIMK proteins (i.e., serine/threonine-protein kinases) play important roles in metastasis by phosphorylating cofilin proteins which are involved in the dynamic remodeling of actin filaments [30]. LIMKs are required for the collective invasion by taking roles in invadopodium formation and extracellular matrix degradation in cancer cells [38,39]. Guo et al. reported a critical increase in LIMK1 levels in HCC, compared to the normal liver tissue. They also showed that the proliferation and migration capacity of liver cancer cells are suppressed with the down regulation of LIMK1 [40]. Another study reported that the inhibition of LIMK/cofilin pathway via PAK1 inhibition (i.e., an

activator of LIMK), suppresses growth of several HCC cell lines, which is accompanied with decreased tumor value in vivo, due to the enhancement of apoptosis in relation to the blocked NF-kB activation [41].

Recent studies have shown that LIMK inhibition is effective in terms of decreasing proliferative and metastatic features various tumor cells [31]. It has also been reported that motility and invasion capacity of breast and prostate cancer cells (with overexpressed LIMK1) was attenuated when inhibitors of upstream LIMK regulators are administered [42]. In the light of this information, LIM kinases can be considered as suitable candidates to target cancer progression and metastasis. …"

5. From the cofilin and wound assays, LIMK1 and LIMK2 seem to have different aspects in liver cancer. It is better to show the secondary protein structure with domains. It also will be necessary to check the genomic aspect or prognostic effects of these two genes from human patient data such as TCGA.

Before explaining our revision to address this item, we first would like to clarify one point regarding the first part of the issue. In text and figures related to cofilin and wound healing assays, we reported the results provided by LIMK inhibitor compounds, given in short as "LIMKi-1", "LIMKi-2", etc. As a result, these reported differences belong to the bioactivities of these inhibitors (i.e., LIMKi-2 was mostly effective, whereas LIMKi-1 was not). Nevertheless, we still examined literature to find statements regarding difference between LIMK1 and LIMK2 in liver cancer (and other cancers). Most of the information was provided over LIMK1, and to the best of our knowledge there was no information specific to LIMK2.

We appreciate the suggestion on the genomic aspect or prognostic effects of LIMKs by our reviewer. Hence, we performed Kaplan-Meier survival analysis in cancer patients in relation to LIMK1 and LIMK2 using TCGA data and discussed the results. We also compared domain annotations and aligned amino acid sequences of LIMK1 and 2. This analysis has been added to section "2.4.1 Selection of Target Proteins" of the manuscript with the text and figures provided below:

"*… With the aim of evaluating the prognostic effects of LIMK1 and LIMK2, we performed a Kaplan-Meier survival analysis on liver cancer (HCC) RNA-seq data, collected from 364 patients [43]. It is observed from the results that the high expression of LIMK1 is significantly associated with lower survival rate in HCC patients (Figure S1a). The median survival rate was calculated as 71 months in patients with low LIMK1 expression; whereas, the mean was 37.8 months in patients with high LIMK1 expression. On the other hand, we did not observe a clear association between the survival rate and LIMK2 gene expression levels (Figure*

S1b). To examine the documented functional and sequence-based similarities/differences between LIMK1 and LIMK2, we checked their domain annotations from the InterPro database, and observed that these proteins have the exact same domain architecture (i.e., from N to C terminal: "IPR001781: Zinc finger, LIM-type" – "IPR001781: Zinc finger, LIM-type" – "IPR001478: PDZ domain" – "IPR001245: Serine-threonine/tyrosine-protein kinase, catalytic domain", detailed information can be obtained from: https://www.ebi.ac.uk/interpro/protein/reviewed/P53667 and https://www.ebi.ac.uk/interpro/protein/reviewed/P53671). A Needleman–Wunsch pairwise global sequence alignment between LIMK1 and LIMK2 (with default parameters of scoring matrix: BLOSUM62, gap open: 10, gap extend: 0.5) showed that these proteins are 51.9% identical and 66.8% similar to each other, and non-similar positions are mainly concentrated around the N and C terminal ends (the full alignment output is given in Supplementary Material section 3). Based on these results, it can be stated that further research is required to analyze different aspects of LIMK1 and LIMK2 in various types of cancer. In the light of these information, we decided to target LIM kinases in the experimental validation part of this study, with the main focus on LIMK1. …"

6. Also, it is better to add an explanation on potential effects or known facts of LIMKs in liver cancer in terms of working mechanisms or any relevant information for liver cancer-specific context.

To the best of our knowledge, LIMKs have not been significantly studied in the context of liver cancer in the literature. As a result, the available information is limited. However, this situation also makes them attractive potential targets for future studies. This issue is related to the one reported in issue number 4, and handled under the same section of the manuscript. To address this issue with the available information from the literature, we added the following text to the section: "2.4 Validation of Predicted Molecular Interactions":

"… Guo et al. reported a critical increase in LIMK1 levels in HCC, compared to the normal liver tissue. They also showed that the proliferation and migration capacity of liver cancer cells are suppressed with the down regulation of LIMK1 [40]. Another study reported that the inhibition of LIMK/cofilin pathway via PAK1 inhibition (i.e., an activator of LIMK), suppresses growth of several HCC cell lines, which is accompanied with decreased tumor value in vivo, due to the enhancement of apoptosis in relation to the blocked NF-kB activation [41]. …"

Also, we added the following sentence with references (to cite the association of LIMKs with different types of cancer including HCC) to the end of the section: "1. Introduction":

"*… As such we analyzed interacting compound predictions of DRUIDom for PI3K/AKT/mTOR pathway proteins, out of which, we focused on LIMK1 and LIMK2 proteins and their new ligand predictions, as these proteins have been associated with several types of cancer especially in terms of progression and metastasis [30-33]. …*"
