# Supplementary material for: Protein domain-based prediction of drug/compound–target interactions and experimental validation on LIM kinases
Source: PLoS Comput Biol. 2021 Nov 29;17(11):e1009171. doi: 10.1371/journal.pcbi.1009171 (PMC8659301; doi:10.1371/journal.pcbi.1009171)
Supplement: Attachment — Submitted filename: PCOMPBIOL-D-21-01053_reviewer_7_20_reviewed.docx [file pcbi.1009171.s009.docx]

1. The assumption behind the mapping between domains and compounds is that either the binding region of the ligand is on the mapped structural domain(s), or there is a functional relationship between the two so that the mapped domain is required for the corresponding bioactivity to occur. (page 7) --> It is not clearly explained how functional relationships can make mapping between domains and compounds. What's the meaning in terms of the biochemistry view?

2. It is not clear on the explanations on coverage extensions on page 11.

3. The Authors should provide statistics on the number of proteins/findings of all pathways not only the PI3K pathway for information to the Readers.

4. It needs more explanation why the Author focused on LIMK among four PI3K pathway proteins.

5. From the cofilin and wound assays, LIMK1 and LIMK2 seem to have different aspects in liver cancer. It is better to show the secondary protein structure with domains. It also will be necessary to check the genomic aspect or prognostic effects of these two genes from human patient data such as TCGA.

6. Also, it is better to add an explanation on potential effects or known facts of LIMKs in liver cancer in terms of working mechanisms or any relevant information for liver cancer-specific context.
